# BMC *Palliative Care* reviewer acknowledgement 2014

**DOI:** 10.1186/1472-684X-14-3

**Published:** 2015-01-24

**Authors:** Catia Cornacchia

**Affiliations:** BioMed Central, Floor 6, 236 Gray’s Inn Road, London, WC1X 8HB UK

## Abstract

The editors of *BMC Palliative Care* would like to thank all our reviewers who have contributed to the journal in Volume 13 (2014).

**Ebun Abarshi**

UK

**Stephanie Aiken**

UK

**Ali Arican**

Turkey

**Antony Arthur**

UK

**Regis Aubry**

France

**Stephen Barclay**

UK

**Raymond Barfield**

USA

**Ingela Berggren**

Sweden

**Parag Bharadwaj**

USA

**Melissa Bloomer**

Australia

**Priska Bützberger Zimmerli**

Switzerland

**Patrick Carragher**

UK

**Yvonne Cartwright**

UK

**Kenneth Chambaere**

Belgium

**Chih-Hung Chang**

USA

**Tai-Yuan Chiu**

Taiwan

**Harvey Chochinov**

Canada

**Katherine Clark**

Australia

**Robin Cohen**

Canada

**Romain Coriat**

France

**Nessa Coyle**

USA

**Joanna Crocker**

UK

**Muhammad Darawad**

Jordan

**Marike De Boer**

Netherlands

**Christopher De Bono**

Canada

**Egidio Del Fabbro**

USA

**Myriam Deveugele**

Belgium

**Ana Draper**

UK

**Lesley Dunleavy**

UK

**Linda Emanuel**

USA

**Kevin Flannelly**

USA

**Kate Flemming**

UK

**Monika Führer**

Germany

**Jan Gaertner**

Germany

**Sheri Gerson**

USA

**Barbara Gomes**

UK

**Laura Goodwin**

UK

**Gunn Grande**

UK

**Kathy Jo Gutgsell**

USA

**Richard Hain**

UK

**Jun Hamano**

Japan

**George Handzo**

USA

**Breffni Hannon**

Canada

**Richard Harding**

UK

**Beth Hardy**

UK

**Dylan Harris**

UK

**Gørill Haugan**

Norway

**Katharina Heimerl**

Austria

**Kirsten Hermans**

Belgium

**Sarah Hoare**

UK

**Jo Hockley**

UK

**Mark Howard**

UK

**Suh-Ing Hsieh**

Taiwan

**Simon Rk Hsieh**

Taiwan

**Peter Hudson**

Australia

**Nic Hughes**

UK

**Sean Hughes**

UK

**Stephanie Jarosek**

USA

**Birgit Jaspers**

Germany

**Bridget Johnston**

UK

**Saskia Juenger**

Germany

**Miguel Julião**

Portugal

**Arif Kamal**

USA

**Kwang-Youn Kim**

USA

**Satomi Kinoshita**

Japan

**Suzanne Kite**

UK

**Jonathan Koffman**

UK

**Berna Komurcuoglu**

Turkey

**Jennifer Kryworuchko**

Canada

**Virginia Lebaron**

USA

**Wojciech Leppert**

Poland

**Deena Levine**

USA

**Bert Leysen**

Belgium

**Maureen Lyon**

USA

**Beth Macgregor**

UK

**Matthew Maddocks**

UK

**Bernd Oliver Maier**

Germany

**Linda Maynard**

UK

**Susan Mcclement**

Canada

**Kenneth Miller**

USA

**Stuart Milligan**

UK

**Geoffrey Mitchell**

Australia

**Mitsunori Miyashita**

Japan

**Toshikazu Moriwaki**

Japan

**Martin Mücke**

Germany

**Cheryl Nekolaichuk**

Canada

**Mary O'Brien**

UK

**Clare O'Callaghan**

Australia

**Karin Oechsle**

Germany

**Amparo Oliver**

Spain

**Bregje D Onwuteaka-Philipsen**

Netherlands

**Evie Papavasiliou**

UK

**Koen Pardon**

Belgium

**Sheila Payne**

UK

**Jamie Penner**

Canada

**Klaus Maria Perrar**

Germany

**Mila Petrova**

UK

**Lara Pivodic**

Belgium

**Kristian Pollock**

UK

**Nancy Preston**

UK

**Ramona Rhodes**

USA

**Judith Rietjens**

Netherlands

**Lenzo Robijn**

Belgium

**Sarah Russell**

UK

**Richella Ryan**

UK

**Yuko Sato**

Japan

**Eva Schildmann**

Germany

**Christian Schulz**

Germany

**David Seamark**

UK

**Lucy Selman**

UK

**Tim Sharp**

UK

**Jessica Simon**

Canada

**Paul Sinfield**

UK

**Aynharan Sinnarajah**

Canada

**Melanie Smith**

USA

**Anna Spathis**

UK

**Carol Stone**

Ireland

**Sayaka Takenouchi**

Japan

**Michael Teut**

Germany

**Alexia Torke**

USA

**Robin Urquhart**

Canada

**Max Vergo**

USA

**Frank Wagner**

Canada

**Xiaojie Wang**

China

**Franca Warmenhoven**

Netherlands

**Donna Wilson**

Canada

**Eleanor Wilson**

UK

**Stefan Wirz**

Germany

**Sriram Yennu**

USA

